# The Phosphoarginine Energy-Buffering System of *Trypanosoma brucei* Involves Multiple Arginine Kinase Isoforms with Different Subcellular Locations

**DOI:** 10.1371/journal.pone.0065908

**Published:** 2013-06-11

**Authors:** Frank Voncken, Fei Gao, Cath Wadforth, Maggie Harley, Claudia Colasante

**Affiliations:** 1 School of Biological, Biomedical and Environmental Sciences, University of Hull, Hull, United Kingdom; 2 Medizinische Zellbiologie, Institut für Anatomie und Zellbiologie II, Justus-Liebig-Universität, Giessen, Germany; Oak Ridge National Laboratory, United States of America

## Abstract

Phosphagen energy-buffering systems play an essential role in regulating the cellular energy homeostasis in periods of high-energy demand or energy supply fluctuations. Here we describe the phosphoarginine/arginine kinase system of the kinetoplastid parasite *Trypanosoma brucei*, consisting of three highly similar arginine kinase isoforms (TbAK1-3). Immunofluorescence microscopy using myc-tagged protein versions revealed that each isoform is located in a specific subcellular compartment: TbAK1 is exclusively found in the flagellum, TbAK2 in the glycosome, and TbAK3 in the cytosol of *T. brucei*. The flagellar location of TbAK1 is dependent on a 22 amino acid long N-terminal sequence, which is sufficient for targeting a GFP-fusion protein to the trypanosome flagellum. The glycosomal location of TbAK2 is in agreement with the presence of a conserved peroxisomal targeting signal, the C-terminal tripeptide ‘SNL’. TbAK3 lacks any apparent targeting sequences and is accordingly located in the cytosol of the parasite. Northern blot analysis indicated that each TbAK isoform is differentially expressed in bloodstream and procyclic forms of *T. brucei*, while the total cellular arginine kinase activity was 3-fold higher in bloodstream form trypanosomes. These results suggest a substantial change in the temporal and spatial energy requirements during parasite differentiation. Increased arginine kinase activity improved growth of procyclic form *T. brucei* during oxidative challenges with hydrogen peroxide. Elimination of the total cellular arginine kinase activity by RNA interference significantly decreased growth (>90%) of procyclic form *T. brucei* under standard culture conditions and was lethal for this life cycle stage in the presence of hydrogen peroxide. The putative physiological roles of the different TbAK isoforms in *T. brucei* are further discussed.

## Introduction

Virtually all eukaryotes contain energy buffering systems for the regulation of their energy homeostasis in periods of high-energy demand or energy supply fluctuations. The majority of these systems depend on phosphagen kinases that catalyse the reversible and ATP-dependent phosphorylation of guanidino acceptor compounds [1], [2]. The derived high-energy phosphagens are relatively small and highly diffusible molecules that provide fast energy supply when energy consumption becomes critical [1]. In addition, they stabilise the cellular ATP/ADP ratio and function as a temporal and spatial energy buffer in the cell [2]. The two most widely distributed energy buffering systems depend on the phosphorylation of creatine or arginine, with the phosphocreatine/creatine kinase system predominantly found in vertebrates and sponges, and the phosphoarginine/arginine kinase system mainly present in invertebrate organisms [1], [2]. Unicellular eukaryotes contain similar energy buffering systems, with phosphoarginine/arginine kinase systems described for the ciliates *Paramecium caudatum* and *Tetrahymena pyriformis*, the choanoflagellate *Monosiga brevicollis*, as well as for some Kinetoplastida [3], [4], [5], [6], [7], [8].

The Kinetoplastida order contains medically and veterinary important protozoa, such as *Trypanosoma brucei, Trypanosoma cruzi* and *Leishmania major* [9]. *T. brucei* is an extracellular parasite and is the causative agent of African trypanosomiasis [10], while the closely related *T. cruzi*, an obligate intracellular parasite, is the causative agent of Chagas disease [11]. Both parasites have a complex life cycle and are transmitted by insect vectors [10], [11]. The life cycle of *T. brucei* alternates between the bloodstream form (BSF) found in the blood and tissue fluids from mammals, and the procyclic form (PCF) in the mid-gut of the tsetse fly. During differentiation and the transition from one host to the other, substantial changes in the energy metabolism take place, which allow adaptation of the parasite to the distinct host environments [12], [13], [14], [15], [16].

Kinetoplastid arginine kinases have so far only been functionally characterised for *T. cruzi* [7], [8]. The genome of *T. cruzi* harbours one functional arginine kinase-coding gene, i.e. TcCLB.507241.30, which is expressed in the different life cycle stages of *T. cruzi* and is exclusively located in the cytosol [7], [8], [17]. Overexpression of TcCLB.507241.30 in *T. cruzi* epimastigotes resulted in an increased survival capacity upon exposure to oxidative challenging compounds such as hydrogen peroxide, suggesting a possible role of this arginine kinase in oxidative stress response [18], [19]. Genome analysis of the closely related kinetoplastid *T. brucei* identified three different arginine kinase-coding genes indicating that its energy buffering system is more complex than the one reported for *T. cruzi* [7], [17], [20]. Here we describe the molecular and functional characterisation of the phosphoarginine/arginine kinase system from *T. brucei*, and discuss its possible function in the parasite energy metabolism.

## Results

### Sequence Analysis

**Figure 1:**
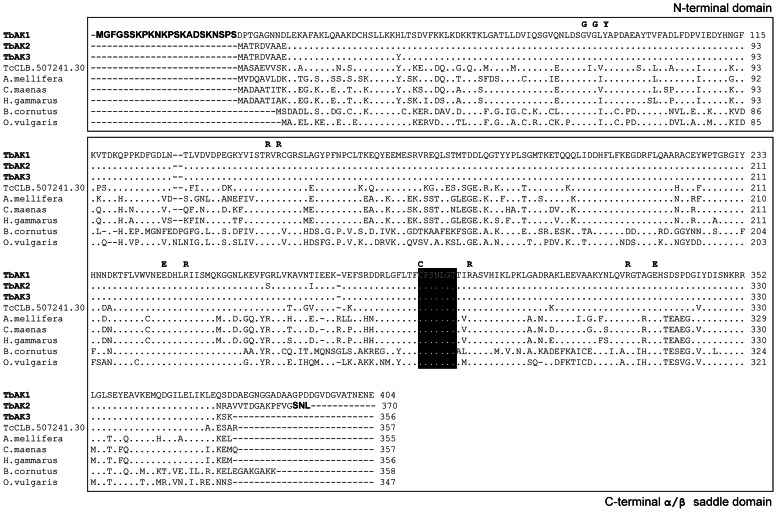
Conserved sequence features of TbAK1-3. Sequence alignment of TbAK1-3, *T. cruzi* TcCLB.507241.30, and representative arginine kinases from insects, crustaceans and molluscs. The N-terminal flagellar targeting signal of TbAK1 and the C-terminal PTS1 signal of TbAK2 are shown in bold face. Key residues involved in the binding of ATP and arginine are indicated on top of the alignment. The phosphagen kinase active site signature sequence is printed white bold face on a black background. The conserved α-helical N-terminal domain and C-terminal α/β saddle domain are boxed. Identical amino acids are represented by a dot and gaps are shown as dashes. See Material and Methods for different organism names and gene accession numbers.

BLASTP sequence analysis, using the *T. cruzi* arginine kinase TcCLB.507241.30 [7] as query, resulted in the identification of three arginine kinase-coding genes in the genome database (GeneDB) of *T. brucei* strain Lister 927, i.e. Tb927.9.6170 (*TbAK1*), Tb927.9.6230 (*TbAK2*) and Tb927.9.6290 (*TbAK3*) [17]. Amino acid sequence alignment (Figure 1) revealed a high degree of similarity between the different trypanosome arginine kinases (85–99%) and selected arginine kinases from invertebrates, including the crustaceans *Carcinus maenas* (86% similarity to TbAK1) and *Homarus gammarus* (86%), and the insects *Drosophila melanogaster* (84%) and *Apis mellifera* (88%). The deduced amino acid sequences of *TbAK1-3* are virtually identical, except for the presence of short sequence extensions at the N- and C-termini of TbAK1 and the C-terminus of TbAK2 (compared to TbAK3, see Figure 1). Sequence analysis showed further that the TbAK1-3 isoforms display a similar protein domain organization as prototypical arginine kinases (Figure 1), including the presence of an α-helical N-terminal domain of about 100 amino acid residues and a >250 amino acids long C-terminal α/β saddle domain [21], [22], [23]. The C-terminal domain contains key residues involved in reaction catalysis, while the N-terminal domain regulates access to the catalytic pocket [21], [22], [23]. Key residues involved in the binding of ATP and the guanidino substrates, as well as the phosphagen kinase active site signature sequence ‘C-P-x(0,1)-[ST]-N-[ILV]-G-T’ (Prosite motif PDOC00103) are conserved in all analysed sequences (Figure 1). The TbAK1-3 isoforms were therefore predicted to function as arginine kinases.

### Arginine Kinase Activity of TbAK1

**Figure 2:**
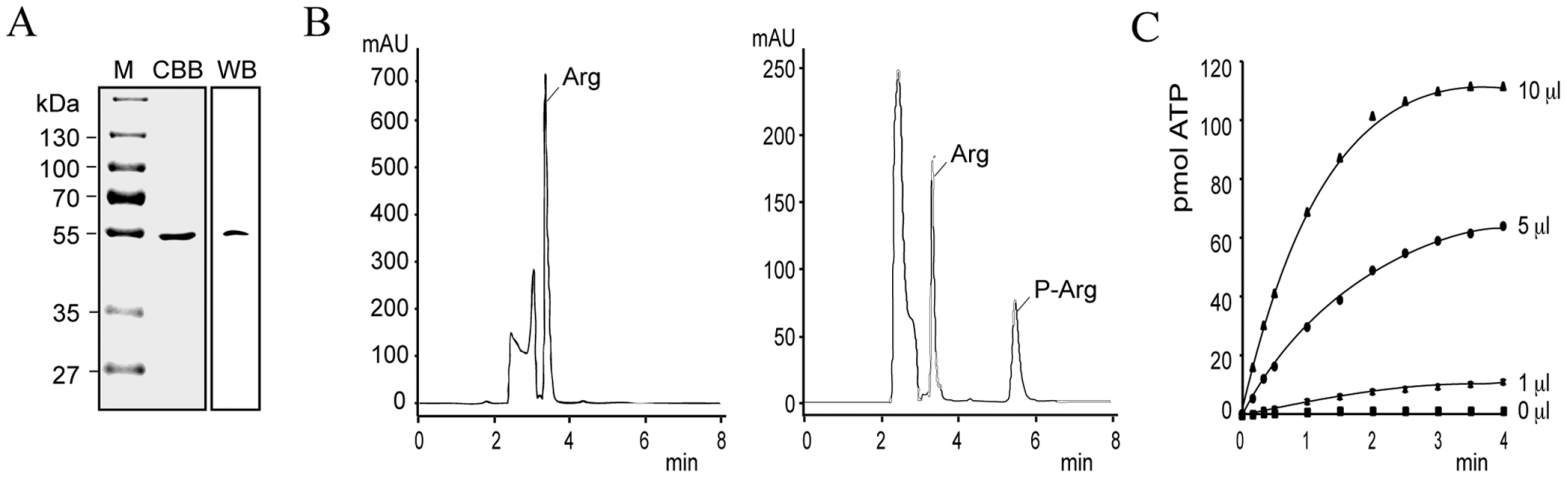
TbAK1-nHis catalyses the reversible phosphorylation of arginine. (**A**) TbAK1-nHis was expressed in *E. coli* BL21 (DE3) and subsequently isolated using Talon affinity chromatography. Left panel: SDS-PAGE and coomassie brilliant blue (CBB) staining revealed a single protein band (∼55 kDa) in the purified TbAK1-nHis protein fraction. Right panel: western blot (WB) analysis using a commercial His-tag antibody stained the same protein band (arrow). (**B**) HPLC analysis of the reaction products formed in the absence (left panel) or presence (right panel) of purified TbAK1-nHis. Elution time of arginine and phosphoarginine are 3.3 min and 5.5 min, respectively. (**C**) Reverse arginine kinase reaction catalysed by recombinant TbAK1-nHis and using ADP (1 mM) and increasing volumes (0–10 µl) of the collected phosphoarginine fraction as substrate. ATP formation from phospharginine was measured using a firefly luciferase-dependent ATP detection kit. Abbreviations are: M, molecular weight marker; kDa, kilodalton.

Recombinant TbAK1 bearing a N-terminal histidine-tag (TbAK1-nHis) was expressed in the heterologous host *Escherichia coli* BL21 (DE3) and subsequently isolated using Talon affinity-chromatography. SDS-PAGE and coomassie brilliant blue staining of the purified TbAK1-nHis protein fraction revealed a single protein band with the expected molecular size of 55 kDa (Figure 2A, left panel). Western blot analysis using a His-tag antibody revealed specific staining of the same protein band (Figure 2A, right panel), thus confirming isolation of the recombinant TbAK1-nHis protein. The arginine kinase activity of the purified TbAK1-nHis protein was first determined in the phosphoarginine synthesis direction at temperatures required for growth of the analysed *T. brucei* life cycle stages, here 28°C for PCF and 37°C for BSF *T. brucei*. The results revealed that TbAK1 catalysed the phosphorylation of arginine with a *V*_max_ of 7.96±0.53 µmol × mg protein^−1^ × min^−1^ at 28°C and 12.26±0.07 µmol × mg protein^−1^ × min^−1^ at 37°C, respectively (Table 1 and Figure S1). We further determined the different *K*_m_ values for the recombinant TbAK1 protein (Table 1 and Figure S1). The arginine concentration was set to 3 mM for determination of *K*_m_^ATP^, while for determination of *K*_m_^arg^ the ATP concentration was set to 0.8 mM. Comparison with published data revealed that the specific activity and *K*_m_^ATP^ of TbAK1 were similar to those previously reported for the recombinant *T. cruzi* arginine kinase, i.e. 10–16 µmol × mg of protein^−1^ × min^−1^ and 0.3 mM, respectively [6]. The pH optimum for TbAK1 was about 8.0 (results not shown). Similar to previously characterized arginine kinases from other eukaryotes, also TbAK1 was specific for arginine and was unable to catalyse the phosphorylation of creatine (not shown).

**Table 1:** *K*_m_ and *V*_max_ values of the TbAK1-nHis arginine kinase activity.

		28°C	37°C
***K*** **_m_**	ATPArginine	0.23±0.030.24±0.05	0.32±0.060.48±0.07
***V*** **_max_**	ATP/Arginine	7.96±0.53	12.26±0.07

*K*_m_ and maximum velocity (*V*_max_) values are shown in mM and µmol × mg^−1^ × min^−1^, respectively.

The synthesis of phosphoarginine by TbAK1 was further analysed by HPLC using a SP2 Hypersil column [24]. The expected elution times for arginine and phosphoarginine are 3.3 and 5.5 min, respectively [24]. Analysis of the resulting elution patterns revealed that in the absence of TbAK1-nHis no phosphoarginine was formed (Figure 2B, left panel), whereas in its presence an additional peak was found with the expected elution time of 5.5 min for phosphoarginine (Figure 2B, right panel). This peak was collected (elution range 5.2–5.8 min) and further analysed using a coupled arginine kinase assay in the direction of phosphoarginine dephosphorylation and ATP synthesis. The enzyme assay mix contained ADP and the eluted HPLC fractions as substrates, as well as the purified recombinant TbAK1-nHis enzyme. ATP formation was measured using a firefly luciferase-dependent ATP detection kit. The results confirmed that the HPLC peak at 5.5 min indeed contained phosphoarginine, since ATP was only synthesized in the presence of TbAK1-nHis and ADP (Figure 2C). As expected, a linear correlation was found between the volume of HPLC eluate used and the amount of ATP produced (Figure 2C). No ATP was generated during control experiments, i.e. where no TbAK1-nHis or heat-inactivated TbAK1-nHis was added during the phosphoarginine synthesis reaction (not shown) or in the absence of HPLC eluate (Figure 2C). These results confirmed that TbAK1 indeed functions as an arginine kinase and catalyses the reversible phosphorylation of arginine.

### Subcellular Location of TbAK1-3

Arginine kinases were previously reported to be associated with different subcellular compartments [3], [4], [5], [25], [26]. The *T. cruzi* arginine kinase TcCLB507241.30 was shown to be exclusively cytosolic, which is in agreement with the absence of identifiable subcellular targeting signals [17]. Sequence alignment revealed that TbAK1 contains distinct N- and C-terminal extensions of respectively 22 and 26 amino acid residues, when compared to TbAK2, TbAK3 and TcCLB507241.30 (Figure 1). Sequence analyses using the SignalP, MITOPROT, Predotar, PSORT and TargetP programs (ExPASy) however failed to attribute any specific subcellular targeting function to these extensions. Also TbAK3, the smallest isoform, did not contain any recognisable subcellular targeting signals (Figure 1). This is in contrast to TbAK2, which contained a conserved type 1 peroxisomal targeting signal (PTS1) at its carboxy-terminal end, i.e. the tri-peptide ‘SNL’ (Figure 1). This tripeptide was previously shown to be sufficient for peroxisomal targeting in eukaryotes [27], [28] and its presence in TbAK2 suggested a glycosomal location in *T. brucei*.

**Figure 3:**
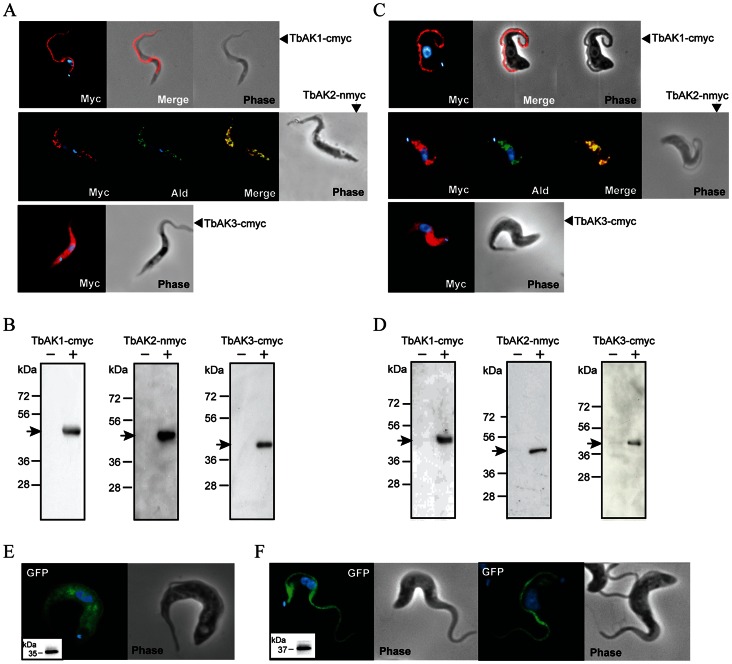
TbAK1-3 isoforms have different subcellular locations in *T. brucei*. Immunofluorescence microscopy of PCF (**A**) and BSF (**C**) *T. brucei* 449 cell lines expressing different myc-tagged TbAK isoforms (red). Glycosomes (green) were detected using a polyclonal antibody directed against the glycosomal marker protein aldolase (Ald) [69], while the nucleus and kinetoplast were stained (blue) with DAPI. The merge (overlay) shows the co-localisation of TbArg2-nmyc and the glycosomal aldolase. Western blot analysis of PCF (**B**) and BSF (**D**) *T. brucei* 449 cell lines grown in the absence (–, non-induced) or presence (+, induced) of tetracycline (0.5 µg.mL^−1^), and using a commercial myc-antibody. For western blot analysis, cells were harvested 24 hours after induction and 2×10^6^ trypanosomes were analysed per gel lane. Abbreviations: Phase, phase-contrast. (**E**, **F**) Immunofluorescence microscopy of PCF *T. brucei* 449 cell lines expressing GFP (E) and NtermTbAK1/GFP (F). GFP is visualized by its natural auto-fluorescence, while the nucleus and kinetoplast were stained (blue) with DAPI. Insets in E and F: western blot analysis using a commercial GFP-antibody confirmed the expression of GFP and the NtermTbAK1/GFP fusion protein.

Because of the high amino acid sequence similarity of TbAK1-3 and the lack of suitable epitopes, no isoform-specific antibodies can be raised. We therefore decided to analyse their subcellular location by expressing recombinant N- or C-terminal myc-tagged protein versions and detecting them using a myc-tag antibody. Western blot analysis confirmed the successful expression of myc-tagged TbAK1-3 in the generated BSF and PCF *T. brucei* cell lines (Figure 3B and 3D, and Figure S2). Immunofluorescence microscopy of the same cell lines revealed that the subcellular localisation of TbAK1 and TbAK2 was dependent on the position of the added myc-tag (Figure 3A and 3C, and Figure S2). TbAK2-nmyc was found exclusively in the glycosomes of PCF and BSF *T. brucei* (Figure 3A and 3C, Figure S3), as expected from the presence of the C-terminal PTS1 signal (see above, and Figure 1). Addition of a myc-tag to the C-terminal end of this arginine kinase (TbAK2-cmyc) resulted in a flagellar location instead (Figure S2). This ‘failure’ to target TbAK2 to the glycosome was anticipated since any sequence addition to a PTS1 signal disrupts its targeting function [29].

Immunofluorescence microscopy of *T. brucei* cell lines expressing TbAK1-cmyc (Figure 3A and 3C, Figure S3) showed that this arginine kinase is exclusively found in the flagellum. Addition of the myc-tag to the N-terminus (TbAK1-nmyc) redirected the protein to the cytosol, suggesting the presence of an N-terminal flagellar targeting signal (Figure S2). We next assessed whether the N-terminal extension of TbAK1 can function independently as a flagellar targeting signal. For this purpose, a PCF *T. brucei* 449 cell line was generated that expressed a GFP-fusion protein (Nterm-TbAK1/GFP) containing the first 22 amino acids of TbAK1 at its N-terminus. A PCF *T. brucei* 449 cell line expressing only GFP was used as a control. Immunofluorescence microscopy revealed that Nterm-TbAK1/GFP was exclusively located in the flagellum (Figure 3F), whereas the native GFP protein was exclusively found in the cytosol (Figure 3E). This result confirmed that the 22 amino acid long N-terminal extension of TbAK1 functions as a flagellar targeting signal in *T. brucei*.

Immunofluorescence microscopy of BSF and PCF cell lines expressing myc-tagged TbAK3 revealed that this protein was located in the cytosol, regardless whether the myc-tag was added to its N- or C-terminus (Figure 3A and 3C, Figure S2 and S3). A cytosolic location of TbAK3 is in agreement with the absence of any identifiable targeting signals in its deduced amino acid sequence.

Overall, our results showed that in *T. brucei* each TbAK isoform is located in a different subcellular compartment.

### Life Cycle Stage-dependent Expression

**Figure 4:**
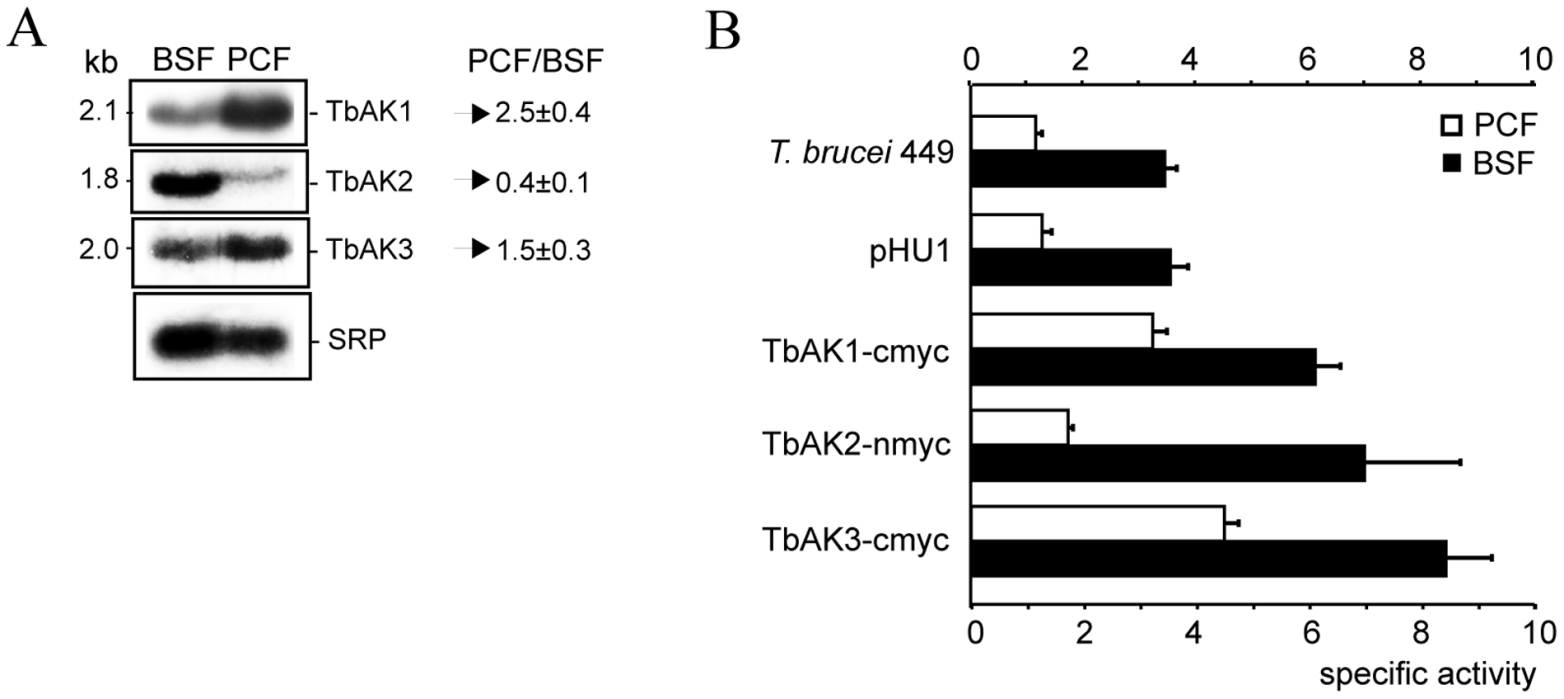
Differential expression of TbAK1-3 in *T. brucei*. (**A**) Northern blot analysis of total RNA (10 µg per lane) isolated from BSF and PCF *T. brucei* 449. The 3′UTRs of *TbAK1-3* were used as DNA probes to detect the respective mRNA transcripts, while the signal recognition particle (SRP) was used as a loading control [32]. Numbers represent the relative mRNA ratios (PCF/BSF) calculated from pixel intensities of the different mRNA bands (ImageJ: http://rsb.info.nih.gov/ij/) after normalisation against SRP, and represent the mean from 3 independent experiments. (**B**) Specific activities of arginine kinase in total cell lysates from the parental (no expression vector), control (containing pHU1 without insert), and myc-tagged TbAK1-3 expressing PCF (white bars) and BSF (black bars) *T. brucei* cell lines. Specific activity (µmol phosphoarginine formed per mg protein per min) was determined in the forward (phosphoarginine synthesis) direction using 2 mM arginine and 0.8 mM ATP as substrates, and using 10 µg of protein (total cell-lysate) per assay. Values are the means of at least three independent experiments.

In *T. brucei,* expression of approximately 2% of genes is developmentally regulated at the mRNA level [30]. Northern blot analysis was used to investigate whether this is also the case for the TbAK isoforms. The open reading frames of *TbAK1-3* display >85% nucleotide sequence identity and can therefore not be used as specific DNA probes. Instead, their highly variable 3′ untranslated regions (UTRs) were used as gene-specific probes. The results revealed a single cross-hybridising mRNA band for each of the *TbAK* genes in both BSF and PCF *T. brucei* (Figure 4A). The observed size-lengths of these mRNAs, here 2.1kb (*TbAK1*), 1.8kb (*TbAK2*) and 2.0kb (*TbAK3*), were similar to the *in silico* predicted mRNA transcript sizes of 2.3kb, 1.8kb and 2.2kb, respectively, calculated using the previously described parameters for the estimation of mRNA transcript lengths in *T. brucei* [31]. The *TbAK* mRNA levels were quantified by hybridizing the northern blots with the signal recognition particle (SRP) DNA probe, which is routinely used for normalisation of mRNA expression levels in *T. brucei* [32]. Quantification revealed that *TbAK1* mRNA is 2.5-fold more abundant in PCF *T. brucei*, *TbAK2* mRNA is 2.8-fold more abundant in BSF *T. brucei*, and *TbAK3* mRNA is 1.5-fold more abundant in PCF *T. brucei* (Figure 4A).

As mentioned above, no isoform-specific antibodies can be raised due to the high sequence similarities of TbAK1-3. This excluded the possibility to assess the expression of the different TbAK isoforms at the protein level. Instead, we measured the total arginine kinase activity in cell extracts derived from the different *T. brucei* cell lines. Comparison revealed substantial differences in the total specific arginine kinase activity depending on the developmental stage of *T. brucei* Lister 449: for the bloodstream form an arginine kinase specific activity of 3.43±0.22 µmol × min^−1^ × mg^−1^ was observed, while for the procyclic form a 3-fold lower specific activity of 1.14±0.12 µmol × min^−1^ × mg^−1^ was found (Figure 4B). Comparison with previously published data showed that the arginine kinase specific activities for PCF *T. brucei* (this paper) were about 4-fold higher than those previously reported for *T. brucei* (unknown procyclic form strain in [33]), and 12-fold higher than those found for *T. cruzi* epimastigotes [33].

### Overexpression, Oxidative Stress and Growth

**Figure 5:**
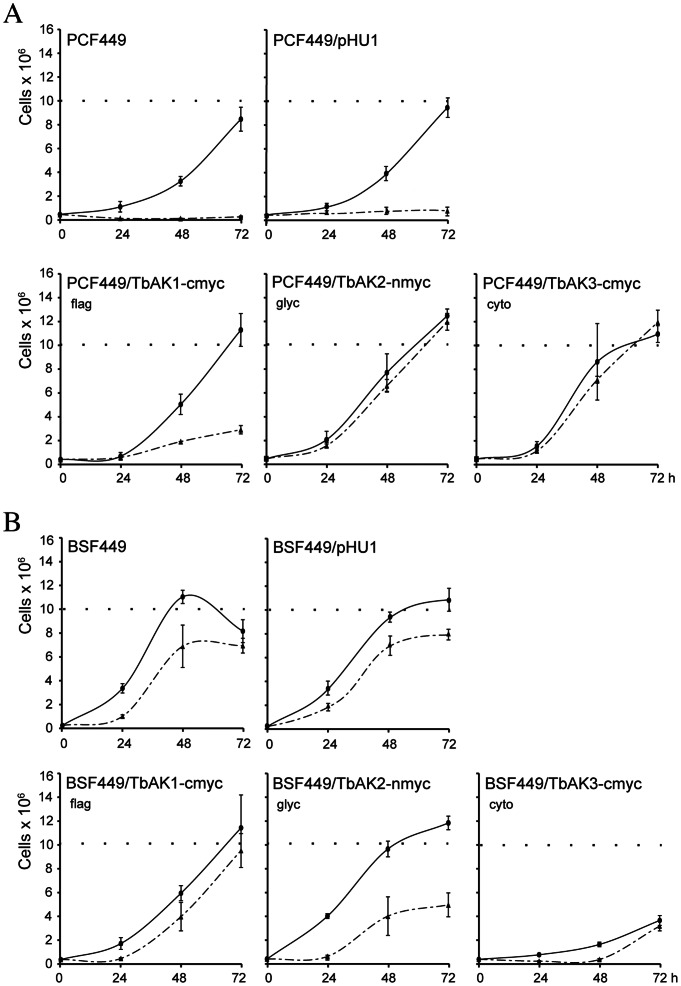
Growth analysis of *T. brucei* 449 cell lines expressing myc-tagged TbAK1-3. Growth curves of parental (PCF449: no vector), control (PCF449/pHU1: containing pHU1 without insert), and myc-tagged TbAK1-3 expressing PCF (**A**) and BSF (**B**) cell-lines under standard (solid lines) and hydrogen peroxide-challenged (dashed lines) culture conditions. Expression of myc-tagged TbAK1-3 was induced by addition of 0.5 µg.ml^−1^ tetracycline 24 hours before the start of the experiment. PCF trypanosomes were oxidatively challenged with 10 µM hydrogen peroxide, while BSF trypanosomes were challenged with 200 µM hydrogen peroxide. Cell densities were determined every 24 hours, for a total of 72 hours. Each growth curve represents the means of >3 independent experiments. Abbreviations: flag, flagellar; glyc, glycosomal; cyto, cytosolic.

Increased expression of TcCLB.507241.30 in *T. cruzi* significantly improved its survival (growth) capacity under standard and oxidative-challenging culture conditions [18]. Here we investigated whether also the TbAK1-3 isoforms play a similar role in *T. brucei.* For this experiment the same cell lines expressing myc-tagged TbAK1-3 were used as for the localisation studies shown in Figure 3A and 3C. The expected higher arginine kinase activity in the TbAK-myc over-expressing cell lines was confirmed by enzyme analysis, which showed an overall ≥1.4-fold increase in total arginine kinase activity in comparison to the corresponding parental and control (containing pHU1 without insert) PCF and BSF *T. brucei* 449 cell lines (Figure 4B). Subsequent growth analysis revealed an overall ≥1.5-fold increase in the growth rates of all PCF TbAK-myc over-expressing cell lines (Figure 5A), suggesting that an increased arginine kinase activity was beneficial to PCF trypanosome growth. For the TbAK-overexpressing BSF *T. brucei* cell lines, however, the growth rates either did not significantly increase (TbAK1-2) or drastically decreased, as shown for TbAK3 (Figure 5B). A higher arginine kinase activity evidently does not benefit growth of the BSF trypanosome, and is even detrimental in case of an increased cytosolic arginine kinase activity.

The same cell lines were subsequently exposed to increasing concentrations of hydrogen peroxide (0–250 µM) in order to assess their capacity to handle oxidative stress. The hydrogen peroxide concentrations required to cause a growth defect were determined empirically for the different cell lines (not shown). Analysis revealed that growth of the corresponding parental and control (containing pHU1 without insert) PCF and BSF *T. brucei* 449 cell lines completely ceased within 24 hours after exposure to 10 µM of hydrogen peroxide (Figure 5A). This growth defect was completely reversed upon the overexpression of glycosomal TbAK2 or cytosolic TbAK3, whereas overexpression of the flagellar TbAK1 resulted in only a partial reversal of this growth defect (Figure 5A). Increasing the hydrogen peroxide concentration to >15 µM was invariably lethal for all analysed PCF cell lines (results not shown). In contrast, BSF *T. brucei* appeared to be far more resistant to oxidative stress than PCF trypanosomes: only a minor growth defect was observed in the presence of 200 µM of hydrogen peroxide (Figure 5B). Addition of 250 µM of hydrogen peroxide was however lethal for all analysed BSF cell-lines (not shown). Notably, the reduced growth of BSF trypanosomes in the presence of 200 µM of hydrogen peroxide could not be reversed by the over-expression of any of the TbAK1-3 isoforms (Figure 5B).

### Silencing of TbAK Expression in PCF *T. brucei* 449

**Figure 6:**
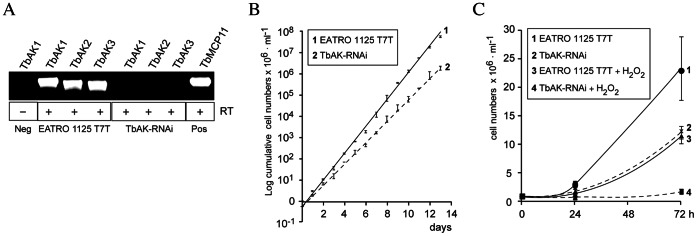
Silencing of TbAK1-3 expression in PCF *T. brucei*. (**A**) TbAK isoform-specific RT-PCR of the parental PCF EATRO1125.T7T cell line (lanes 2–4) and the derived *TbAK-RNAi* cell line (lanes 5–7) cultured for 6 days in the presence of tetracycline (0.5 µg.ml^−1^). No TbAK1 PCR-product was found in the absence (–) of reverse transcriptase (lane 1), indicating that the mRNA samples were not contaminated with genomic DNA. PCR of the non-related gene TbMCP11 (lane 8, [44]) was used as a positive control for cDNA synthesis. (**B**) Cumulative growth curves of the PCF EATRO1125.T7T cell line (solid line) and the tetracycline-induced *TbAK-RNAi* cell line (dashed line) showed an about 100-fold difference in cell density after 12 days under standard culture conditions. (**C**) Growth curves of the parental PCF EATRO1125.T7T cell line (solid lines) and the derived *TbAK-RNAi* cell line (dashed lines), grown under standard and oxidative challenging culture conditions. RNAi in the PCF *TbAK-RNAi* cell line was induced with tetracycline for 6 days prior to the start of the experiment, ensuring complete silencing of all TbAK isoforms (see (A)). For the oxidative-challenging culture conditions, 0.025 µM of hydrogen peroxide was added to the medium.

Whether arginine kinase activity is essential for the growth of *T. brucei* was assessed by RNA interference (RNAi). For this purpose, a tetracycline-inducible PCF *TbAK-RNAi* cell line was generated, which allowed the simultaneous silencing of all three TbAK isoforms. The *TbAK1-3* mRNA levels were monitored by RT-PCR using isoform-specific PCR primers. The results for the tetracycline-induced PCF *TbAK-RNAi* cell line revealed that no PCR product could be detected after 6 days of tetracycline induction (shown in Figure 6A), which suggested a major decrease in *TbAK* mRNA levels compared to the parental EATRO 1125 T7T cell line. Accordingly, no arginine kinase activity could be detected when analysing cell extracts from the induced *TbAK-RNAi* cell line. This result confirmed the successful silencing of TbAK1-3 expression. Growth analysis revealed that under standard culture conditions the growth rate of the TbAK-depleted cell line had decreased to about 10% of the parental EATRO 1125 T7T cell line (Figure 6B). Moreover, addition of 0.025 µM hydrogen peroxide to the TbAK-depleted cell line was found to be lethal, while growth of the parental cell line decreased to about 50% of its original capacity (Figure 6C). Overall, the TbAK-depleted cell line became >400-fold more sensitive to hydrogen peroxide. Our results confirmed that arginine kinase activity is required for the growth of PCF *T. brucei* under standard culture conditions, and is essential for the parasite under oxidative stress conditions.

## Discussion

Phosphagens like phosphoarginine are highly diffusible and metabolically inert molecules that can be stored in the cell without affecting the overall ATP/ADP balance, a critical factor for the regulation of many metabolic processes [1], [2], [5]. Phosphagens are therefore the molecules of choice for the transport and buffering of energy in highly polarised eukaryotic cells [5]. Phosphagen kinases are often located near sites of high ATP turnover, allowing the transfer of energy from a source of ATP production to a site of ATP consumption [1], [2]. Several models have been postulated in which the spatial (subcellular) distribution of phosphagen kinases plays a crucial role in intracellular energy transport as well as in the control and regulation of ATP-dependent enzymes and pathways in the different subcellular compartments [2]. Also the energy metabolism of *T. brucei* is highly compartmentalised, with ATP-producing pathways located in the glycosome, mitochondrion and cytosol [13], [16], [34]. The contribution of these subcellular compartments to the overall ATP production is dependent on the developmental stage of the parasite [13], [16], [34]. ATP production in BSF *T. brucei* depends primarily on substrate-level phosphorylation during glycolysis, which takes place in part in a specialised peroxisome called the glycosome, and the cytosol [34]. This is in contrast to PCF *T. brucei,* which primarily depends on the mitochondrial breakdown of proline and concomitant substrate-level and oxidative phosphorylation for its ATP production [13], [16].

We provided evidence that *T. brucei* expresses 3 different arginine kinase isoforms, i.e. TbAK1-3, with each TbAK isoform located in a different subcellular compartment: TbAK3 is exclusively cytosolic, while TbAK1 and TbAK2 are located in the flagellum and glycosome, respectively. The deduced amino acid sequences of TbAK1-3 are virtually identical, except for the presence of short sequence extensions at the N- and C-termini of TbAK1 and the C-terminus of TbAK2. We have shown here that the flagellar location of TbAK1 is dependent on a specific N-terminal flagellar signal sequence, whose targeting function was disrupted by the addition of a myc-tag. Localisation studies using GFP fusion proteins revealed that the first 22 amino acids of TbAK1 were sufficient to target GFP to the trypanosome flagellum. Putative N-terminal targeting sequences have previously been proposed for other flagellar *T. brucei* proteins, but their exact amino acid sequence composition remains elusive [35], [36], [37]. Extensive sequence analysis and comparison of the TbAK1 N-terminal sequence to those from other flagellar *T. brucei* proteins did not reveal any sequence similarities (not shown), suggesting that it represents a novel trypanosome flagellar targeting signal.

The glycosomal location of TbAK2 is supported by the presence of a conserved C-terminal PTS1 signal, i.e. the tripeptide ‘SNL’. Furthermore, the protein was previously detected during LC-MS analysis of highly purified *T. brucei* glycosomes [38]. Addition of a myc tag to the C-terminus of TbAK2 resulted in ablation of the glycosomal (peroxisomal) targeting, and TbAK2-cmyc was now located in the flagellum of *T. brucei*. Such disruption of a PTS1 signal by sequence extension was previously shown to abolish peroxisomal targeting in other eukaryotes (29). Why TbAK2-cmyc was re-routed to the flagellum is unclear. One possible explanation could be that it contains a flagellar targeting signal in addition to the C-terminal PTS1 signal. TbAK2 however lacks the N-terminal extension present in TbAK2, which was shown here to function as a flagellar targeting signal. The only difference between TbAK2 and the smallest cytosolic TbAK3 isoform is the presence of a short 14 amino acid long sequence extension preceding the C-terminal PTS1 signal. Comparison of this amino acid sequence to known flagellar targeting signals did however not reveal any sequence similarity (not shown), and its potential function as such remains to be experimentally determined. Another possible explanation for the re-routing of TbAK2-cmyc to the flagellum could be oligomerisation with a protein containing a flagellar targeting signal. Such ‘piggy-backing’ protein transport mechanism has been reported previously for various peroxisomal, glycosomal, glyoxysomal and vacuolar proteins [39], [40], [41]. Notably, arginine kinases can form heterodimers [42], and a possible heterodimer formation of the flagellar TbAK1 with TbAK2-cmyc could be an explanation for the observed flagellar localisation of TbAK2 upon ablation of its glycosome-targeting signal. Whether such heterodimer formation indeed occurs in *T. brucei* has to be further investigated.

Overexpression of any of the TbAK isoforms in PCF and BSF *T. brucei* resulted in a significant increase in total cellular arginine kinase activity (see Figure 4B). This result, and the virtual identical amino acid sequences of the different isoforms, indicate that also TbAK2 and TbAK3 function as arginine kinases, in a similar fashion as shown here for the purified TbAK1 enzyme. Though a similar catalytic activity is suggested for TbAK1-3, the specific subcellular location of each isoform might indicate differences in their respective physiological function(s). ATP production and/or consumption in each subcellular compartment of *T. brucei* are dependent on the lifecycle stage of the parasite [13], [16], [34]. Northern blot analysis revealed that expression of the glycosomal TbAK2 is 2.8-fold upregulated in BSF *T. brucei* 449. This result correlates well with the crucial role of the glycosome in the BSF energy metabolism [34]. However, the general accepted model is that there is no net ATP synthesis in this organelle, with glycolysis-derived ATP primarily produced in the cytosol [34]. If no net ATP-production occurs in the glycosome, why would this organelle then require a TbAK2-dependent energy buffering system? The *T. brucei* glycosome further contains several adenylate kinases, which were proposed to play a role in the maintenance of the glycosomal ATP/ADP balance and the removal of AMP by the reversible conversion of ATP and AMP to 2 molecules of ADP [43]. The presence of both adenylate kinase and TbAK2 in BSF and PCF glycosomes implicates that under certain conditions either a surplus or deficit of ATP can exist in this organelle, which again could be counter-balanced by the presence of a phosphoarginine/arginine kinase energy buffering system.

Northern blot analysis revealed further a significantly up-regulated expression of the cytoplasmic TbAK3 (1.5-fold) and flagellar TbAK1 (2.5-fold) in PCF *T. brucei* 449, suggesting a more prominent role of both TbAK isoforms in this life cycle stage of the parasite. The majority of the ATP produced in PCF *T. brucei* is derived from its mitochondrion [13], [16]. The produced ATP is exported from the mitochondrion to the cytosol via the ATP/ADP-carrier TbMCP5 [44], [45]. In the cytosol, part of the ATP could be converted to phosphoarginine by the cytoplasmic TbAK3 to serve as a highly mobile cellular energy carrier and local energy buffer. The specific subcellular location of TbAK1 indicates that this arginine kinase is most probably involved in the energy homeostasis of the flagellum. The *T. brucei* flagellum contains further enzymes that are normally associated with the mitochondrial and glycosomal energy metabolism, such as enolase, pyruvate kinase, phosphoglycerate mutase, and adenylate kinase [43], [46], [47]. Also these enzymes were proposed to function in the maintenance of the flagellar energy homeostasis [43], [48]. The *T. brucei* flagellum plays an essential role in cell motility as well as cell division [49]. Flagellar mobility consumes significant quantities of ATP and provision of the more bulky ATP across the *T. brucei* flagellum is considered to be rather difficult due to the flagellar length, compact protein structure, and the narrow connection between flagellum and cytosol [48], [50], [51]. The alternative channelling of phosphagens, instead of the more bulky ATP, between mitochondrion and flagellum was previously described for spermatozoa of the sea urchin *Strongylocentrotus purpuratus*, with mitochondrial and flagellar creatine kinases representing respectively ATP source and ATP sink [52]. Also the phosphoarginine system of *P. caudatum* was reported to be involved in the supply of energy to the cilia [4]. Here, we propose a similar spatial energy-buffering system for *T. brucei,* with the cytosolic TbAK3 acting as a phosphoarginine source and the flagellar TbAK1 providing ATP to fuel flagellar motility.

Another important function of the phosphagen/phosphagen kinase system is the provision of energy in periods of increased energy-demand or during energy fluctuations [1], [2]. Increased expression of TcCLB.507241.30 in *T. cruzi* epimastigotes or the heterologous expression of the same phosphagen kinase in either *Escherichia coli* or *Saccharomyces cerevisiae* resulted in a substantially increased resistance to oxidative challenging compounds [18], [53], [54]. Vice versa, an increased cellular arginine kinase activity was measured upon exposure of *T. cruzi* epimastigotes to hydrogen peroxide [19]. These findings suggested an important role of the phosphoarginine/arginine kinase system in oxidative stress response, a process that inherently consumes substantial amounts of ATP and eventually can lead to ATP-depletion [55], [56]. Also the different developmental stages of the exclusively extracellular parasite *T. brucei* are constantly exposed to oxidative stress, predominantly as a result of the innate immune response of its different hosts, but also as a consequence of its own unusual, partly aerobic fermentative energy metabolism [57], [58], [59], [60], [61]. In particular BSF *T. brucei* lives in an environment, i.e. the mammalian bloodstream, in which oxidative stress factors are highly abundant [58]. Measurement of total arginine kinase activity indeed revealed a substantially higher specific arginine kinase activity in BSF *T. brucei*, which correlated well with its increased capacity (about 20-fold) to withstand oxidative stress. Similar to *T. cruzi*, a substantially increased oxidative stress resistance was observed upon the increased expression of the different TbAK isoforms in PCF *T. brucei*. Vice versa, PCF *T. brucei* became highly sensitive to oxidative stress upon RNAi and complete depletion of its arginine kinase activity. Overexpression of TbAKs in BSF *T. brucei*, however, did not result in the expected increase in oxidative stress resistance, but lead to an overall inhibition of trypanosome growth under standard culture conditions. A possible explanation for this phenomenon could be that an increased expression of arginine kinase in BSF *T. brucei* disturbs its cellular ATP/ADP ratio.

This study has raised a number of important questions, which require further investigation. For example, why does *T. brucei* express 3 different arginine kinase isoforms with specific subcellular localisations, whereas the related kinetoplastid *T. cruzi* contains only a single cytoplasmic arginine kinase? What is the contribution of each TbAK isoform to the spatial energy-buffering system of *T. brucei*? Is each of the different TbAK isoforms required for trypanosome survival (growth) and/or differentiation? Further research will focus on the depletion of the individual TbAK isoforms by gene deletion in the different life cycle stages of *T. brucei* in order to gain more insight into their subcellular compartment-specific physiological roles.

## Materials and Methods

### Gene Accession Numbers and Sequence Alignment

GeneDB and NCBI gene data base accession numbers of the different arginine kinase sequences used for sequence alignment are: TcCLB.507241.30 [*T. cruzi*]; TbAK1, Tb927.9.6170 [*T. brucei*]; TbAK2, Tb927.9.6230 [*Trypanosoma brucei*]; TbAK3, Tb927.9.6290 [*Trypanosoma brucei*]; C.maenes, AF167313 [*Carcinus maenas*]; H.gammarus, CAA48654.1 [*Homarus gammarus*]; A.mellifera, NP_001011603.1 [*Apis mellifera*]; D.melanogaster, NP_729446.1 [*Drosophila melanogaster*]; B.cornutus, BAA22870.1 [*Batillus cornutus*]; and O.vulgaris, BAA95609.1 [*Octopus vulgaris*]. Sequence alignment was obtained using ClustalW2 [62], which was manually optimized using the Sequence Alignment Editor v2.0a11 program (Se-Al; http://tree.bio.ed.ac.uk/software).

### Expression and Purification of Recombinant TbAK1-nHis Enzyme

The open reading frame of *TbAK1* (Tb927.9.6170) was PCR-amplified using the sense primer 5′-ggacggcatatgggcttcggatcatcaaaacc-3′ and the antisense primer 5′gcttgcaggatccttcattttcattcgtgg ctacaccgtctac-3′. The PCR product was cloned into the bacterial expression vector pET16b (Invitrogen) using the restriction sites *Nde*I and *Bam*HI included in the primer sequences (underlined). Expression from pET16b results in the addition of 10 N-terminal histidine residues to the protein. The resulting plasmid was used for the transformation of *E. coli* BL21 (DE3). Protein expression was induced in TB medium supplemented with 10 mM malate and 10 mM pyruvate at 37°C by addition of 1 mM IPTG at an OD_600_ of 0.4. The IPTG-induced culture was grown for 3 hours, after which the bacteria were harvested by centrifugation at 5000×g for 15 minutes. The cell pellet was resuspended in Talon-Binding and Washing Buffer containing 50 mM Na-Phosphate pH 8.0, 300 mM NaCl, 0.01% (v/v) Tween-20, and protease inhibitor (Roche), lysozyme (200 µg ml^−1^) and DNaseI (10 µg ml^−1^), followed by incubation for 30 minutes at 4°C. Cells were lysed using a French Press, and the cell-rests and eventual intact cells were spun down for 15 minutes at 5000×g and 4°C. The supernatant was incubated for 30 minutes at 4°C with magnetic Talon Dynabeads, pre-equilibrated in Talon-Binding and Washing Buffer according to the manufacturer’s protocol (Dynal Biotech). The supernatant was removed and the Dynabeads were washed 5 times with Binding and Washing Buffer. TbAK1-nHis was finally eluted using Talon Elution Buffer (150 mM Imidazole, 50 mM Na-Phosphate buffer pH8.0, 300 mM NaCl, and 0.01% (v/v) Tween-20).

### Measurement of Arginine Kinase Activity

For the measurement of total cellular arginine kinase activity, cell lysates were prepared by re-suspending 1×10^8^ trypanosomes in 200 µl of 50 mM Tris-HCl pH 7.5 and performing 2 subsequent freeze-thawing cycles. Arginine kinase activity was measured in the forward direction (phosphoarginine formation) using an established coupled enzyme assay [63]. The assay mixture contained: total trypanosome cell lysate (10 µg protein) or purified TbAK1-nHis protein, 50 mM Tris-acetate pH 8.6, 5 mM magnesium acetate, 50 mM ammonium acetate, 0.75 mM phosphoenolpyruvate, 0.45 mM NADH, 0.8 mM ATP, and 2 mM arginine. The assay mixture was incubated at either 37°C or 28°C, and the reaction was started by the addition of pyruvate kinase (5 units) and lactate dehydrogenase (5 units). NADH consumption was measured using a spectrophotometer at 340 nm: for each mole of NADH consumed, one mole of phosphoarginine is produced.

### Phosphoarginine Synthesis and Analysis

Phosphoarginine was synthesized using the forward arginine kinase reaction (see above) in the presence of purified TbAK1-nHis. Phosphoarginine formation was analysed by HPLC as described previously [24]. The used HPLC configuration consisted of an Agilent 1100 HPLC system including an ALS injector, a DAD detector, a quaternary pump and a fraction collector. Samples were separated on a SP2 Hypersil column (250 mm × 4.6 mm, 5 µm particle size) in combination with a Phenomenex guard column containing an ID NH2 cartridge (4 × 3.0 mm). Separation was isocratic at 25°C using 72% (v/v) 20 mM phosphate buffer (pH 2.6) and 28% (v/v) acetonitrile as mobile phase and a flow rate of 1 ml min^−1^. The DAD detector was set at 205 nm, with the reference set to 360 nm. 50 µl of arginine kinase reaction mixture were injected per run. The phosphoarginine-containing peak, with an expected retention time of 5.5 minutes [24], was collected and freeze-dried to remove acetonitrile. The obtained residue powder was solved in 100 µl ultrapure H_2_O and subsequently analysed for the presence of phosphoarginine using the reverse arginine kinase coupled enzyme reaction. The reverse arginine kinase reaction mixture contained: variable amounts of phosphoarginine peak fraction, 50 mM Tris-acetate pH 8.6, 5 mM magnesium acetate, 50 mM ammonium acetate and 1 mM ADP. The reaction was started by addition of 5 µg of the purified TbAK1-nHis enzyme and the formation of ATP was quantified *in situ* using the ATP Bioluminescence CLS II Assay Kit (Roche Applied Science) and a Junior LB9509 tube luminometer (Berthold Technologies).

### Trypanosome Cell Culture

The parental proyclic form (PCF) and bloodstream form (BSF) *Trypanosoma brucei* 449 cell lines were cultured in MEM-PROS [64] and HMI-9 [65] medium, respectively. During routine culture, phleomycin (PCF, 0.5 µg ml^−1^; BSF, 0.2 µg ml^−1^) was added to the culture medium to maintain stable expression of the tetracycline repressor from plasmid pHD449 [66]. The parental PCF cell line EATRO1125.T7T was routinely cultured in SDM79 medium in the presence of G418 (10 µg ml^−1^) and hygromycin (25 µg ml^−1^) to maintain stable expression of the tetracycline repressor and T7 RNA polymerase from plasmids pLew90/Neo and pHD328 [67]. All media were supplemented with 10% (v/v) foetal calf serum (Sigma-Aldrich).

### Western Blot Analysis

Trypanosome cells were harvested at mid-logarithmic phase densities of 1×10^6^ cells ml^−1^ (BSF) and 1×10^7^ cells ml^−1^ (PCF) for protein analysis. 2×10^6^ trypanosomes were pelleted for each lane, resuspended in denaturing SDS-containing Laemmli buffer, and the sample denatured for 5 min at 95°C. Proteins were separated on a denaturing 12% sodium dodecylsulfate-containing polyacrylamide gel (SDS-PAGE), and subsequently transferred to a Hybond-P membrane (GE Healthcare Life Sciences) in transfer buffer (39 mM glycine, 48 mM Tris-base, 20% (v/v) methanol, pH 8.3) for 1 h at 100 V. The membrane was blocked by a 30 min incubation at room temperature in Tris–buffered saline (TBS) containing 0.2% (v/v) Tween 20 (TBS-T) supplemented with 7.5% (w/v) non-fat dry milk with gentle shaking, and subsequently incubated for 1 h in TBS-T containing 7.5% (w/v) milk and the primary antibody (diluted 1∶1000). The membrane was then washed once for 15 min, and twice for 5 min in TBS-T, followed by incubation for 45 min at room temperature with the respective secondary antibody (GE Healthcare Life Sciences). The membrane was washed once for 15 min, and four times for 5 min in TBS-T. Finally, the membrane was processed according to the manufacturer’s protocol of the ECL detection kit, followed by exposure to ECL-film (GE Health Care Life Sciences).

### Northern Blot Analysis

*T. brucei* cells were harvested at mid-logarithmic phase densities of 1×10^6^ cells ml^−1^ (BSF) and 1×10^7^ cells ml^−1^ (PCF) RNA analysis. Total RNA was isolated from BSF and PCF *T. brucei* strain 449 using the RNeasy Mini Kit (Qiagen). RNA was separated on a denaturing formaldehyde-containing agarose gel, and transferred to a Hybond-N membrane (GE Healthcare). The different *TbAK* 3′-UTRs (300 bp downstream of stop codon) were used as DNA-probes for hybridisation. [^32^P]dCTP-labelled DNA probes were PCR-amplified using 5′-ctaggatccggtgagggtgatgcattgtt-3′ (sense) and 5′-ctagggccctgaacaaccctacaaaatctctctc-3′ (antisense) for *TbAK1*; 5′-atcggatccgttc tttcctttctttcatacatttcc-3′ (sense) and 5′-atcgggcccagaggattcgaaaagcgtaatcggaacagttg-3′ (antisense) for *TbAK2*; and 5′-ggacggaagcttaccatggctacccgcgacgttgctgc-3′ (sense) and 5′-gcttgcaggatcccttcgacttctccagtttgatgagct caag-3′ (anti-sense) for *TbAK3*. RNA blots were pre-hybridized in hybridization solution (5xSSC, 5xDenhardt’s reagent, and 0.5% (w/v) sodium dodecyl sulphate (SDS)) for 1 h at 65°C, followed by the addition of the [^32^P]dCTP-labelled DNA probes and overnight hybridization at 65°C. The blots were washed at 65°C in subsequently 1x SSC (0.15 M NaCl, 0.015 M sodium citrate) supplemented with 0.1% (w/v) SDS and 0.1xSSC supplemented with 0.1% (w/v) SDS, followed by exposure to X-ray film (Kodak).

### Expression of Myc-tagged TbAK1-3

The open reading frames of *TbAK1-3* were PCR-amplified using genomic DNA from *T. brucei* strain 449 as template, and the primers 5′-ggacggaagcttac catgggcttcggatcatcaaaacc-3′ (sense) and 5′-gcttgcaggatccttcattttcattcgtggctacaccgtctac-3′ (antisense) for *TbAK1*; 5′-ggacggaagcttaccatggcta cccgcgacgttgctgc-3′ (sense) and 5′-gcttgcaggatcccaagttcgaaccaacaaatggtttggcgc-3′ (antisense) for *TbAK2*; and 5′-ggacggaagcttaccatggctacccgcgacgttgctgc-3′ (sense) and 5′-gcttgcaggatcccttcgacttc tccagtttgatgagctcaag-3′ (antisense) for *TbAK3*. The restriction enzyme sites *Hin*dIII (sense primers) and *Bam*HI (antisense primers) are underlined and were used for subsequent cloning into the trypanosome expression vectors pHU1 or pHU2 [44]. Comparison of the cloned *TbAK1-3* sequences from *T. brucei* strain 449 with the sequences of the corresponding loci in the genome sequence database (GeneDB) of *T. brucei* strain 927 revealed only a few sequence differences at the DNA level, but none in the predicted amino acid sequences. The *T. brucei* expression vectors pHU1 and pHU2 are derived from pHD1700 and pHD1701 [44], respectively, and contain next to the unique *Not*I restriction enzyme cleavage site two additional unique sites, i.e. *Pme*I and *Bcl*I, which can be used for linearization of the plasmids and subsequent trypanosome transfection. Tetracycline-inducible expression from these vectors will result in the addition of a 2xmyc tag to either the C-terminal (pHU1) or N-terminal (pHU2) end of the expressed protein. PCF and BSF *T. brucei* 449 cell lines constitutively expressing the tet-repressor (*TETR BLE*) were transfected with the resulting pHU1 and pHU2 constructs containing *TbAK1-3*, as well as with the empty pHU1 vector (used as control). Hygromycin resistant clonal cell lines were isolated as described previously [66]. Trypanosome clones bearing a tetracycline-inducible and 2xmyc-tagged ectopic copy of the *TbAK1-3* isoforms were subsequently analysed by western blotting using a commercial myc antibody (Roche Applied Science). Expression of the different N-terminal (nmyc) or C-terminal (cmyc) myc-tagged TbAK isoforms was induced by the addition of tetracycline (0.5 µg ml^−1^).

For growth analysis, PCF and BSF cultures were diluted to a density of respectively 0.5×10^6^ cells ml^−1^ and 0.2×10^6^ cells ml^−1^ at the start of the experiment. Culture media were supplemented with phleomycin (PCF, 0.5 µg ml^−1^; BSF, 0.2 µg ml^−1^) and hygromycin (PCF, 50 µg ml^−1^; BSF, 15 µg ml^−1^) to provide the same (selective) growth conditions for all cell lines. Expression of the different myc-tagged TbAK isoforms was induced by addition of tetracycline (0.5 µg ml^−1^) to the culture medium 24 hours prior to the start of the growth experiment. During the growth experiment, tetracycline was added daily to all cell lines to maintain expression of the recombinant myc-tagged isoforms and to maintain similar culture conditions. To analyse growth under oxidative stress conditions, trypanosomes were cultured in the presence of hydrogen peroxide, here 10 µM for PCF and 200 µM for BSF cell lines. Cell densities were determined every 24 h using a Neubauer haemocytometer.

### RNA Interference

Expression of all *TbAK* isoforms was down regulated (silenced) in PCF *T. brucei* by RNA interference, using the conserved central part of *TbAK1-3* as target sequence. The primers 5′-ggaagcttgacgttgatccggaaggtaa-3′ and 5′-cactcgagctcttcattcacccacacgag-3′ were used for PCR amplification of the 342 bp sense sequence and the primers 5′-caggatccgacgttgatccggaaggtaa-3′ and 5′-cgaagctttccctcgagttcactgccttcacaagacg-3′ were used for the corresponding 398 bp antisense sequence. The resulting sense and antisense *TbAK* sequences were cloned into pLew100, using the unique restriction enzyme sites (underlined) included in the primers [67]. The resulting *pLew100-TbAK* RNAi construct contains a phleomycin resistance gene, with the consecutively cloned sense and antisense *TbAK* target sequences separated by a 50 bp spacer sequence. Inducible expression is under control of the procyclic acidic repetitive protein (PARP) promoter linked to a prokaryotic tetracycline (tet) operator. The *pLew100-TbAK* RNAi construct was used for transfection of the PCF *T. brucei* EATRO1125.T7T cell line [67]. The *TbAK-RNAi* cell line was obtained after clonal selection in SDM79 medium containing hygromycin (25 µg ml^–1^), neomycin (10 µg ml^−1^) and phleomycin (5 µg ml^−1^).

Tetracycline (0.5 µg ml^−1^) was added to the *TbAK-RNAi* cell line to induce RNA interference and the concomitant down-regulation (silencing) of TbAK1-3 expression. Depletion of the different TbAK isoforms was assessed at the mRNA level by reverse transcriptase-directed cDNA synthesis and PCR. For this purpose, total RNA (30 µg per sample) was isolated, which was subsequently treated with DNase to remove contaminating genomic DNA. The reaction was stopped by addition of 8 mM EDTA, followed by an incubation for 20 minutes at 75°C. mRNA was converted to cDNA by using an oligo(dT)18 primer, Maxima Reverse Transcriptase and in the presence of Ribolock RNase inhibitor as described in the manufacturer’s protocol (Fermentas). The reverse transcriptase reaction was stopped by incubation at 85°C for 5 minutes. The above described primer combinations were used for the specific PCR-amplification of the different TbAK1-3 isoforms.

For growth analysis, cultures of the tetracycline-induced PCF *TbAK-RNAi* cell line and the parental EATRO1125.T7T were diluted to a density of respectively 0.5×10^6^ cells ml^−1^ at the start of the experiment. During the growth experiment, selective antibiotics were omitted from and the inducer tetracycline added to all culture media to provide comparable growth conditions for all cell lines. To analyse growth under oxidative stress conditions, cell lines were cultured in the presence of 10 µM hydrogen peroxide. Cell densities were determined every 24 h using a Neubauer haemocytometer.

### Functional Analysis of the TbAK1 Flagellar Targeting Signal

The complete open reading frame of GFP was PCR-amplified from plasmid pHD1595 (C. Clayton, University of Heidelberg, Germany) using 5′-gcaggatccatggtgagcaagggcg-3′ (sense) and 5′-gctattaattaaggacttgtacagctcg-3′ (antisense) as primers. The restriction sites *Bam*HI and *Pac*I (underlined) included in the primers were used for the cloning of GFP into the expression vector pHU1. The resulting pHU1-GFP construct allows the tetracycline-inducible expression of GFP. The primers 5′-gctgtttaaactgatcaccaatttcactacaacg-3′ (sense) and 5′-cgtggatcctgatggagaattctttgaatcagctt tggacg-3′ (antisense) were used to PCR-amplify a 932 bp DNA fragment from pHU1-TbAK1, including the first 66 bp of the *TbAK1* ORF (coding for the putative N-terminal targeting signal at its 3′ end. The PCR primers include *Pme*I and *Bam*HI restriction sites (underlined) that were used for the subsequent cloning of this DNA fragment into pHU1-GFP construct, thereby replacing the original 866 bp *Pme*I and *Bam*HI DNA fragment. Sequencing of the resulting pHU1-NtermTbAK1/GFP construct confirmed the in-frame cloning of the putative TbAK1 flagellar targeting signal and GFP. The resulting pHU1-GFP and pHU1-NtermTbAK1/GFP constructs were used for transfection of PCF *T. brucei* 449, and hygromycin resistant cell lines were isolated by clonal selection as described previously [66]. Expression of GFP or the NtermTbAK1/GFP fusion protein was induced by the addition of tetracycline (0.5 µg ml^−1^) and was confirmed by western blot analysis using a commercial GFP antibody (Roche Applied Science).

### Microscopy

PCF and BSF trypanosomes were centrifuged from culture medium at 2,000×g and resuspended in phosphate-buffered saline (PBS) containing 4% (w/v) paraformaldehyde. Fixed cells were allowed to settle down and attach to poly-L-lysine-coated microscope slides. Staining of trypanosomes with 4′,6′-diamidino-2-phenylindole (DAPI, DNA-staining) and labelling (immunofluorescence) with the different antibodies (see Figure legends) was performed as previously described [38], [44], [68]. GFP was visualised in paraformaldehyde-fixed cells by recording its natural green auto-fluorescence. Cells were examined using a Leica DM RXA digital de-convolution microscope and images recorded using a digital camera (Hamamatsu).

## Supporting Information

Figure S1 Lineweaver-Burk plots used for the determination of the different Michaelis-Menten constants, here *K*_m_^ATP^ and *K*_m_^arg^, and the maximum velocity (*V*_max_) for the phosphoarginine synthesis catalysed by TbAK1-nHis at respectively 28°C and 37°C. Arginine kinase activity was measured in the forward phosphoarginine producing direction. Values and error bars are derived from >3 independent experiments.
(TIF)

Figure S2 Immunofluorescence microscopy of PCF (**A**) and BSF (**C**) *T. brucei* 449 cell lines expressing myc-tagged TbAK1-3 (red). The nucleus and kinetoplast were stained (blue) with DAPI. Western blot analysis of PCF (**B**) and BSF (**D**) cell lines grown in the absence (–, non-induced) or presence (+, induced) of tetracycline (0.5 µg ml^−1^), and using a commercial myc-antibody. For western blot analysis, cells were harvested 24 hours after induction and 2×10^6^ trypanosomes were analysed per gel lane. Abbreviations: Phase, phase-contrast; kDa, kilodalton.
(TIF)

Figure S3 Immunofluorescence microscopy (fields of cells) of PCF and BSF *T. brucei* 449 cell lines expressing different myc-tagged TbAK isoforms (red). Glycosomes (green) were detected using a polyclonal antibody directed against the glycosomal marker protein aldolase (Ald) [69], while the nucleus and kinetoplast were stained (blue) with DAPI. The merge (overlay) shows the co-localisation of TbAK2-nmyc and the glycosomal aldolase.
(TIF)
