# Supplementary figures and images for: The Phosphoarginine Energy-Buffering System of Trypanosoma brucei Involves Multiple Arginine Kinase Isoforms with Different Subcellular Locations
Source: PLoS One. 2013 Jun 11;8(6):e65908. doi: 10.1371/journal.pone.0065908 (PMC3679164; doi:10.1371/journal.pone.0065908)

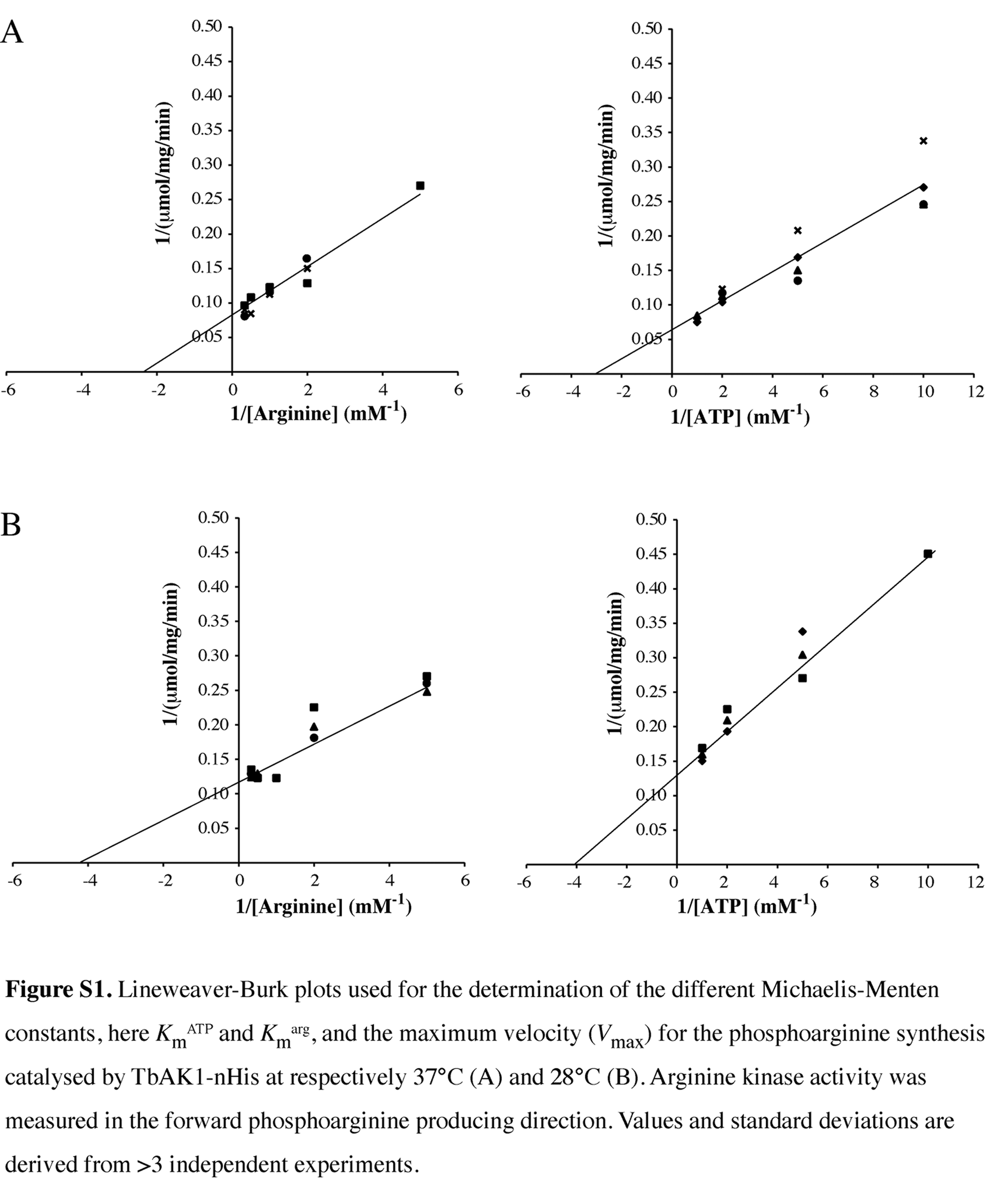

Supplement: Figure S1 — Lineweaver-Burk plots used for the determination of the different Michaelis-Menten constants, here K m ATP and K m arg, and the maximum velocity (V max) for the phosphoarginine synthesis catalysed by TbAK1-nHis at respectively 28°C and 37°C. Arginine kinase activity was measured in the forward phosphoarginine producing direction. Values and error bars are derived from >3 independent experiments. (TIF) [file pone.0065908.s001.tif]

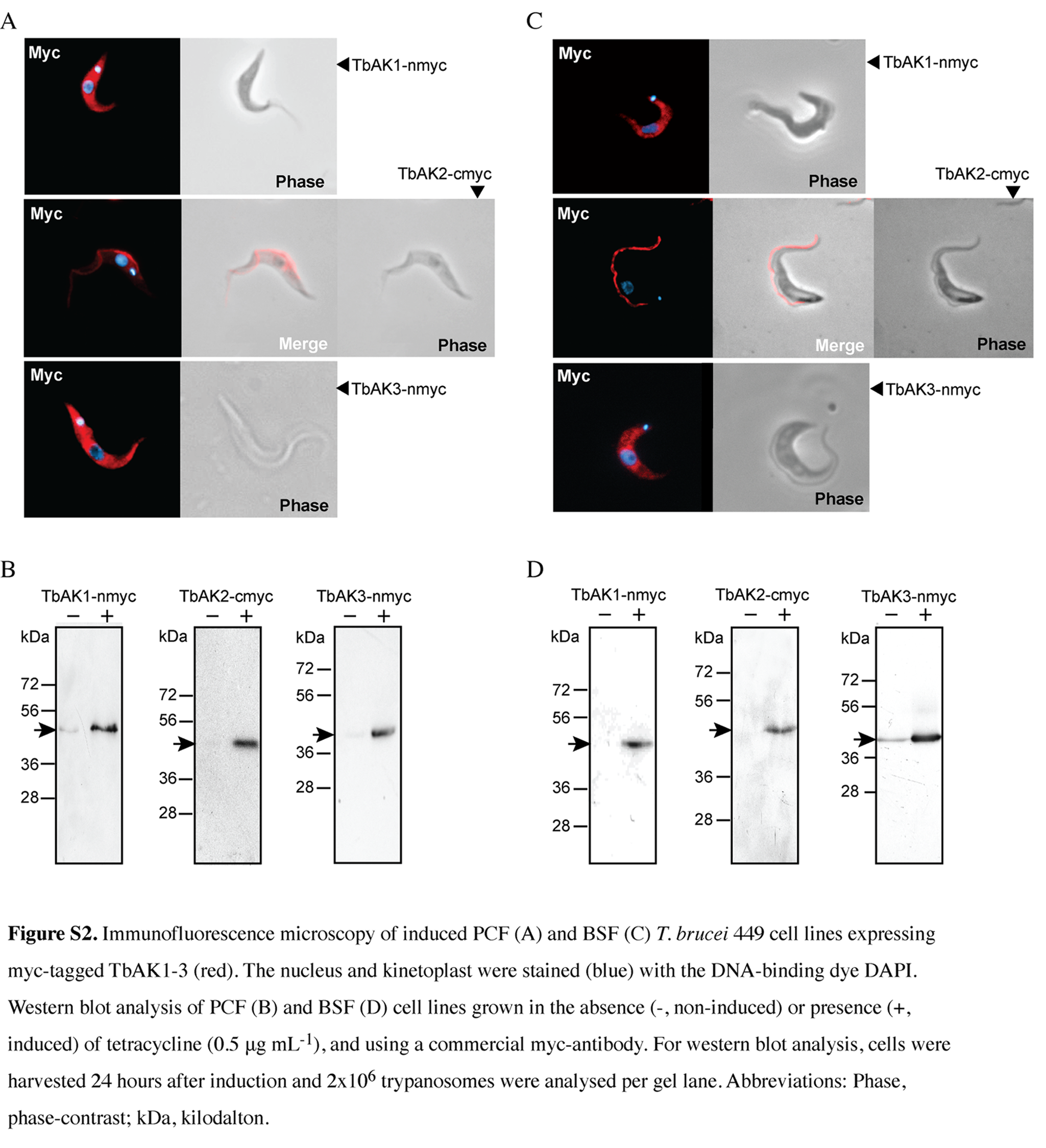

Supplement: Figure S2 — Immunofluorescence microscopy of PCF (A) and BSF (C) T. brucei 449 cell lines expressing myc-tagged TbAK1-3 (red). The nucleus and kinetoplast were stained (blue) with DAPI. Western blot analysis of PCF (B) and BSF (D) cell lines grown in the absence (–, non-induced) or presence (+, induced) of tetracycline (0.5 µg ml−1), and using a commercial myc-antibody. For western blot analysis, cells were harvested 24 hours after induction and 2×10^6^ trypanosomes were analysed per gel lane. Abbreviations: Phase, phase-contrast; kDa, kilodalton. (TIF) [file pone.0065908.s002.tif]

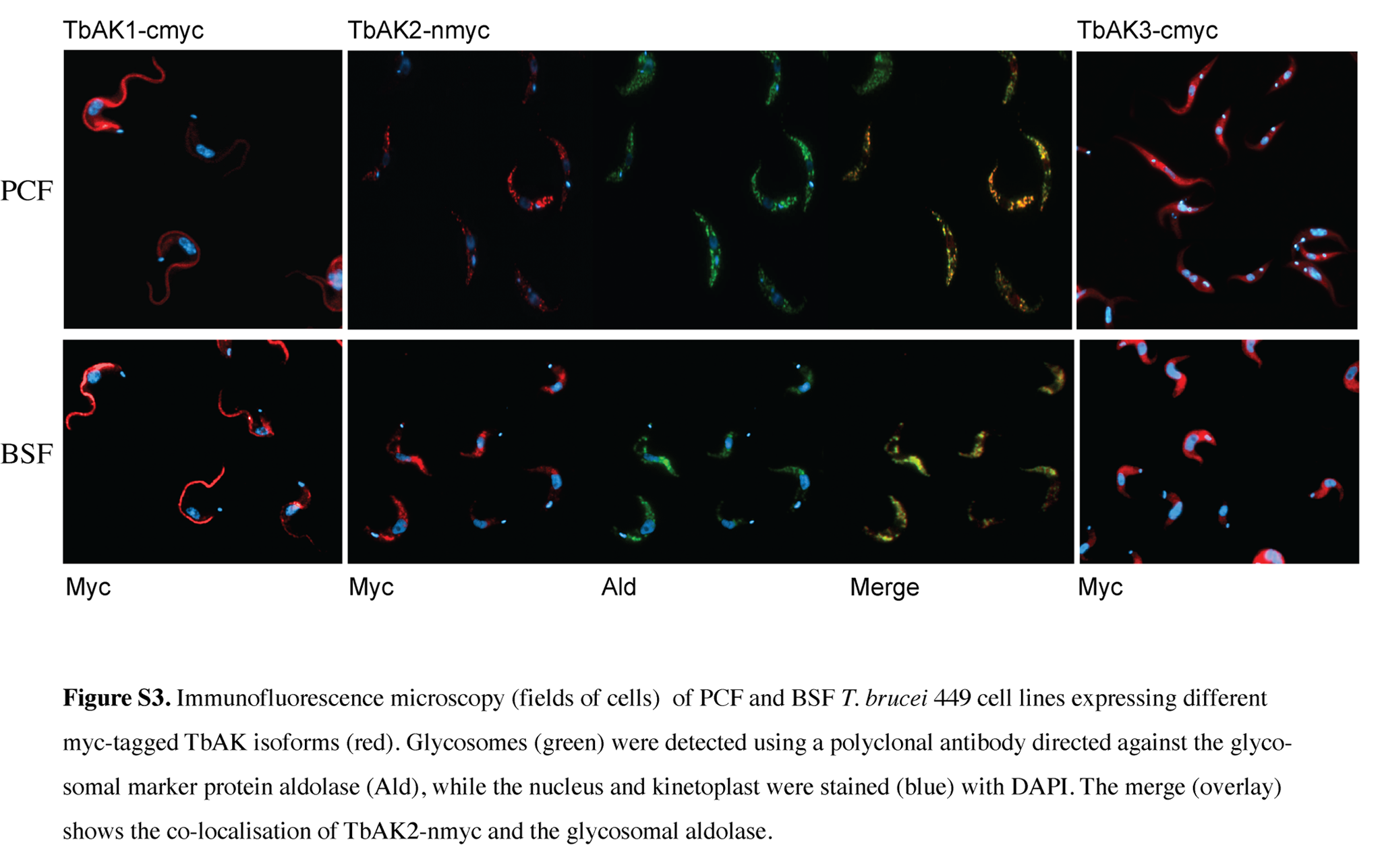

Supplement: Figure S3 — Immunofluorescence microscopy (fields of cells) of PCF and BSF T. brucei 449 cell lines expressing different myc-tagged TbAK isoforms (red). Glycosomes (green) were detected using a polyclonal antibody directed against the glycosomal marker protein aldolase (Ald) [69], while the nucleus and kinetoplast were stained (blue) with DAPI. The merge (overlay) shows the co-localisation of TbAK2-nmyc and the glycosomal aldolase. (TIF) [file pone.0065908.s003.tif]
